# Overcoming barriers for the adoption of Local Energy and Flexibility Markets: A user-centric and hybrid model

**DOI:** 10.1016/j.jclepro.2021.128323

**Published:** 2021-10-01

**Authors:** Guntram Pressmair, Evgenia Kapassa, Diego Casado-Mansilla, Cruz E. Borges, Marinos Themistocleous

**Affiliations:** a e7 Energy Innovation & Engineering, Walcherstrasse 11, 1020 Vienna, Austria; b Department of Digital Innovation, University of Nicosia, 28th October 24, 2414, Nicosia, Cyprus; c Faculty of Engineering, University of Deusto, Avenida de las Universidades 24, 48007 Bilbao, Spain; d Deusto Institute of Technology, Faculty of Engineering, University of Deusto, Avenida de las Universidades 24, 48007 Bilbao, Spain

**Keywords:** Local Flexibility Market, Local Energy Market, Market model, Barriers detection, Delphi method

## Abstract

To achieve the European climate targets and the Paris Agreements, at least 65% of the electricity needs to be generated from renewable energy sources by 2030. This requires a significant increase of distributed energy resources, posing a challenge for distribution system operators to integrate them into existing hierarchical grids. The concept of Local Flexibility Markets has recently gained attention as a market-based tool to tackle this challenge, making use of demand side flexibility. In this paper a Delphi method has been performed, showing that there are still numerous barriers in place preventing a widespread adoption of such markets in Europe. The main obstacles for market participants refer to standardisation issues. Based on that, a hybrid market model has been developed, comprising elements of a Local Flexibility Market and a Local Energy Market. To activate demand side flexibility from local energy transactions, spatio-temporally varying price signals are introduced, reflecting the constraints of the distribution grid. The paper shows, that this novel market approach helps to overcome relevant standardisation issues, but also certain barriers regarding end-users’ lifestyles, which is because prices are comprehensible signals that can motivate end-users to participate. Moreover, a set of numerical examples is provided to illustrate the monetary benefits that could be gained by consumers and prosumers in the proposed hybrid market model. The examples show that the major share of the cost savings result from local energy trading, but the hybrid market model is also able to accumulate additional smaller revenues from providing flexibility. Finally, the systematic approach of characterising the market model in this paper offers a valuable framework for other researchers to map their ideas among existing approaches of Local Energy and Flexibility Markets.

## Highlights

• A framework for characterising Local Energy and Flexibility Markets is provided.
• Main barriers for adopting such markets are related to standardisation issues.
• A hybrid market model is proposed, featuring local energy and flexibility trading.
• Trades are steered by dynamic price signals, reflecting various flexibility needs.
• Numerical examples indicate the expected financial implications.

## Abbreviations

AS: Ancillary Services
BaU: Business as Usual
BRP: Balance Responsible Party
CEC: Citizen Energy Community
CEER: Council of European Energy Regulators
COO: Chief Operating Officer
DER: Distributed Energy Resource
DLMP: Distribution Locational Marginal Pricing
DSF: Demand Side Flexibility
DSO: Distribution System Operator
ECSP: Energy Community Service Provider
ESCO: Energy Service Company
EU: European Union
EV: Electric Vehicle
HV: High Voltage
LEM: Local Energy Market
LEMO: Local Energy Market Operator
LFM: Local Flexibility Market
LFMO: Local Flexibility Market Operator
LV: Low Voltage
MO: Market Operator
MV: Medium Voltage
P2P: peer to peer
PBC: Price Based Control
PV: Photovoltaics
REC: Renewable Energy Community
RES: Renewable Energy Sources
SESP: Smart Energy Service Provider
TLC: Traffic Light Concept
TSO: Transmission System Operator
WS: Wholesale

## 1 Introduction

### 1.1 Motivation

In late 2020, the European Union (EU) updated its climate targets with the aim to reach overall carbon neutrality by 2050 (European Commission, 2020). According to that, the new targets for 2030 foresee a reduction of CO_2_ emissions of 55% compared to 1990. For the power system this means that by 2030 at least 65% of the electricity will be generated from Renewable Energy Sources (RES). An increasing share of these RES will be connected to the distribution grid. Therefore, grid integration of Distributed Energy Resources (DERs) poses a major concern for Distribution System Operators (DSOs), who are responsible for the security and stability of the local grid (Al-Shetwi et al., 2020). This is because DERs include fluctuating distributed generators (solar panels and wind mills) and novel loads such as Electric Vehicles (EVs) (Zou et al., 2020), which may lead to increased violations of voltage limits and congestions due to high power flows. In order to cope with these challenges, DSOs among other stakeholders in the electricity system require Demand Side Flexibility (DSF). The use of DSF is a promising approach, offering business opportunities for a range of market actors and also end-users, who increasingly emerge to so-called prosumers (Hamwi et al., 2020). To activate DSF on a local level, several market-based instruments have been discussed in the literature recently. This paper draws a clear distinction between Local Energy Markets (LEMs) and Local Flexibility Markets (LFMs), with the aim to propose a hybrid market model combining elements of both concepts.

On the one hand, an LEM can be defined as a market platform for trading locally generated (renewable) energy peer to peer (P2P) among residential agents (Weinhardt et al., 2019), which usually aims to maximise the local consumption and enable prosumers to manage their energy surplus and deficit independently from fixed feed-in tariffs. On the other hand, LFMs aim to provide a marketplace for the DSO to procure flexibility and avoid voltage violations and congestion (Jin et al., 2020).

From the perspective of the Council of European Energy Regulators (CEER) (CEER, 2018), a market-based procurement procedure is the preferred option for efficiently activating flexibility for the DSO, making LFMs attractive concepts. Also, the EU Clean Energy Package introduces renewable energy communities (EU, 2018) and citizen energy communities (EU, 2019) as legal entities. According to that, energy communities can facilitate various new services such as community generation, aggregation, P2P trading or charging services, which creates a promising environment for future LEMs and LFMs.

### 1.2 Scope and structure of the paper

This work has been carried out within the Horizon 2020 PARITY project (PARITY, 2020) and it is an extension of a previous conference paper by the authors, in which they identified the barriers that hinder the adoption of LFMs and LEMs (Zabaleta et al., 2020). The aim of the PARITY project is to develop a local blockchain-based market at distribution network level that is able to solve congestion and voltage problems of the DSO, but at the same time empowers end-users to play an active role in the electricity system.

To this end, the aim of the current paper is to propose the high level hybrid market model of PARITY, specifying the interaction of market participants, the coordination mechanisms and its integration with existing electricity markets. Moreover, this paper discusses the strengths and weaknesses of this hybrid model, which combines elements of both LEMs and LFMs. Additionally, the paper analyses to which extent the novel approach fits the needs of the participants involved and if it is able to overcome some of the previously identified barriers.

The systematic approach of characterising the PARITY hybrid market model in this paper should furthermore serve as a guide for other researchers to frame their ideas in the context of the previous work regarding LEMs and LFMs. Without going into too much detail on further implementation steps, this high-level market design approach might help to trigger the development of novel market-based frameworks in the scientific community.

The scientific discussion on LEMs and LFMs is vivid, with also some hybrid models already being proposed. However, it seems there is a lack of models that are user-centric and also comprehensible for the end-users. Addressing this gap, the proposed hybrid market model makes use of DSF and enables end-users to participate in local P2P transactions. The novelty of this approach lies in the fact that the P2P transactions are steered through additional price signals reflecting the flexibility needs of the DSO. In this way, end-users face a clear and transparent price signal incentivising both grid-friendly load profiles and increased consumption of locally generated renewable energy.

The remainder of the paper is organised as follows: After a brief description of the methodology applied (Section 2), Section 3 starts with a review of related research projects and specifies the research gap addressed in this paper. Continuing the literature review, the key parameters for characterising a new market model are identified and reviewed. In Section 4, the results of the applied Delphi method are presented, prioritising barriers for the widespread adoption of LEMs and LFMs, and identifying conflicts of interests in such new schemes, from the perspective of energy market participants. Then, the proposed hybrid and user-centric market model is introduced in Section 5, while in Section 6 the authors provide a set of numerical examples as a preliminary validation indicating the order of magnitude of the expected financial implications. Finally, in Section 7 the authors discuss their findings, highlighting the strengths and weaknesses of the proposed market model. The article concludes by providing key take-aways and an outlook for future work in Section 8.

## 2 Methodology

**Fig. 1:**
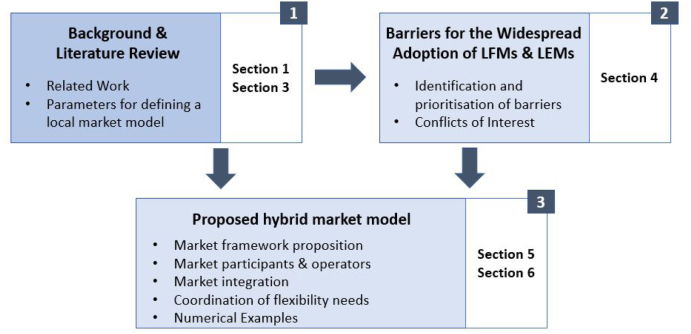
Followed methodology.

In this section, the followed methodology is described to propose the novel hybrid market model. To do so, the authors followed a three-step methodology as depicted in Fig. 1.

Firstly, a literature review was conducted. The authors made a thorough review on previous work proposing local markets in the electricity system, in order to specify the research gap addressed in this paper. The literature review continues by introducing key parameters to characterise such a market model, including (a) the purpose, classifying the energy and flexibility services that can be delivered, (b) instruments to achieve this purpose in a market-based manner, (c) controversies about the definition of the local scope of such a market, (d) market participants and possible market operators, (e) the integration of the local market with existing markets and (f) the need for a prioritisation and coordination of flexibility services.

Secondly, the authors identified and prioritised the barriers that hinder the adoption of local energy and flexibility markets and also highlight a set of conflicts of interests among the different roles present in such markets. These are the results of a consultation process with stakeholders, through the Delphi approach. The Delphi method (Linstone et al., 1975) is based on the fact that group decisions tend to be more accurate than individual decisions. This approach is an iterative procedure designed to help a panel of experts to reach consensus about a topic, consisting of the following steps: (a) A panel of experts is assembled, (b) forecasting tasks are set and distributed to the experts, (c) experts return initial forecasts and justifications, (d) aggregated inputs are analysed by the coordination team, (e) aggregated feedback is delivered to the experts who have to review their initial forecasts in light of the results of the aggregated information provided, and (f) step “d” and “e” are repeated until consensus is made.

Finally, at the third step, the authors proposed the design of a hybrid market model (LEM and LFM), addressing the prioritised barriers, the conflicts of interest and aiming to bridge the identified gap. The expected financial implications are illustrated in a set of numerical examples, where a small local market of residential prosumers and consumers is modelled in an optimisation problem.

## 3 Literature review

### 3.1 Related work

A useful approach for classifying projects in this field is applied by Ibn Saif and Khadem (2020). They differentiate three categories depending on the product that is traded: (a) LEMs, where energy is traded locally, (b) flexibility markets, where services are offered to energy actors such as DSOs, Transmission System Operators (TSOs) or Balance Responsible Partys (BRPs), which includes LFMs and (c) hybrid models that address both energy and flexibility trading.

One of the first projects testing a LEM under real-life conditions has been the US-based Brooklyn Microgrid project (Mengelkamp et al., 2018). It comprises a blockchain-based market platform for trading surplus electricity from local sources without the need for a central intermediary. Primarily, the existing grid is used for energy supply, but also a separate physical micro-grid can be used to run the LEM in island mode. In Europe, a prominent concept for innovative local P2P energy trading is NRGcoins (Mihaylov et al., 2018). Based on the blockchain technology, NRGcoins introduces a virtual currency to remunerate injections and withdrawals of energy from the grid. It is decoupled from the energy retail business and the aim is to balance local production and consumption by remunerating surplus feed-in only if it can be consumed locally in near real time. There are also a range of initiatives where the Market Operator (MO) of the LEM acts as an energy supplier, balancing the whole portfolio. Examples for such concepts are Piclo (UK) and Vandebron (Netherlands) (Zhang et al., 2017).

Four of the most relevant large scale trials of LFMs are NODES, GOPACs, Enera and Piclo Flex (Schittekatte and Meeus, 2020). In all of these market platforms it is intended that several DSOs can procure flexibility. NODES is operated by the power exchange Nordpool and represents a marketplace where the flexibility products are specified by the location of the flexible asset. In this way, DSOs can procure local flexibility and the offers not needed on a local level are forwarded to other markets such as the intraday and balancing market. Trials are currently running in Norway and Germany (NODES, 2018). Similarly, Enera is operated by the power exchange EPEX Spot and tested in Northern Germany, but it is designed as a platform exclusively for flexibility procurement of TSOs and DSOs. Piclo Flex is even more specific focusing on flexibility requests from DSOs only with currently six DSOs participating. As a different approach, GOPACS is not an actual market platform but an intermediary between the needs of network operators (DSOs, TSOs) and the Dutch intraday Wholesale (Wholesale (WS)) market platform ETPA (Schittekatte and Meeus, 2020). The Danish iPower project proposes a flexibility clearing house (FLECH), which is an independent non-profit entity clearing and settling offers from aggregators with bids from DSOs. The FLECH market is designed as running parallel to existing WS and Ancillary Services (Ancillary Services (AS)) markets (Zhang et al., 2014). Similarly to iPower, the EcoGrid 2.0 project establishes a market where DSOs procure flexibility from aggregators, running in parallel with existing markets. However, on the EcoGrid 2.0 platform both scheduled (without capacity reservation) and conditional (with capacity reservation) flexibility products can be traded (Heinrich et al., 2020). In contrast,  Esmat et al. (2018) formulate a flexibility market that is operated by the DSO itself and takes place after the day-ahead WS market. In the INVADE project (Olivella-Rosell et al., 2018b), the flexibility MO role is assumed by an aggregator and also BRPs are enabled to buy flexibility services on this local market. Opening up the LFM for other participants than the DSO is also proposed by Minniti et al. (2018), where the needs of WS market agents are also addressed in the local market.

Finally, some hybrid market models tackle both energy and flexibility trading on local level. In the DOMINOES project, prosumers are enabled to trade energy among peers as well as towards an aggregator for WS and AS market participation. This platform is operated by the Energy Community Service Provider (ECSP), a role which could be assumed by an independent aggregator or a BRP (Mendes et al., 2019). The EMPOWER project follows a similar approach and introduces the Smart Energy Service Provider (SESP) as a MO, that offers combined contracts for energy and flexibility trading (Olivella-Rosell et al., 2018a).

Although there have been already proposed some hybrid models in the literature that combine elements of LEMs and LFMs, the authors observed a lack of approaches that integrate the monetary value of flexibility for the DSO with the pricing mechanisms of the P2P energy trading. By pricing in the grid state in a dynamic way, end-users can face a single price signal that reflects both the local energy and flexibility needs.

### 3.2 Parameters for defining a local market model

#### 3.2.1 Purpose

The fundamental parameter to be defined is the purpose the local market should serve. For the products and services that are delivered through the market, a differentiation between energy and flexibility can be made.

From a product perspective, electrical energy can be referred to as a commodity that can be used by end-users for operating electric devices. Similarly, flexibility can be seen as a commodity, characterised by Villar et al. (2018): (a) direction (up or down), (b) amount of capacity and/or power, (c) starting time and duration, (d) response time, (e) location (in the transmission or distribution grids).

From a service perspective, energy and flexibility tackle different needs. A suitable definition is given by USEF (Klaassen and Van der Laan, 2019): Energy services “(potentially) affect the amount of energy consumed or produced” by a prosumer, whereas flexibility services “focus on deliberate (time limited) changes to the ‘normal’ energy profile”. Based on this differentiation, the following energy and flexibility services can be provided by prosumers through a local market:

• **Energy services** include
  1. P2P supply with locally generated electricity among local prosumers (Klaassen and Van der Laan, 2019).
  2. Selling of locally generated renewable electricity to a centralised energy supplier. In most countries, this is supported by subsidised feed-in-tariffs (Yu-zhuo et al., 2017).
• **Flexibility services** include (USEF, 2015):
  1. Flexibility services for the DSO with the overall aim to avoid or delay grid reinforcement:
    – Congestion management by reducing peak loads at specific nodes in the distribution grid to avoid thermal overload of system components.
    – Voltage control by increasing or decreasing load or generation to avoid violating voltage limits.
  2. Flexibility services for the TSO:
    – Frequency control by increasing or decreasing load or generation in the area served by a TSO to maintain system stability. There are organised markets for primary, secondary, and tertiary control.
    – Congestion management by reducing peak loads at specific nodes in the transmission grid.
  3. Flexibility services for BRPs, which are related to portfolio optimisation and aim at reducing sourcing costs.

#### 3.2.2 Market-based instruments

The concepts of LEMs and LFMs can serve as market-based instruments to provide these energy and flexibility services.

In an LEM, prosumers can sell their surplus electricity production and other local consumers can buy this surplus by increasing their loads or replacing centrally sourced electricity in their load profile (Siano et al., 2019). The intention of LEMs is to give consumers the opportunity to make more informed choices about how they buy and sell electricity and to become active participants in electricity markets (Bray, 2018). By trading energy in an LEM, prosumers can achieve certain cost savings. Firstly, these savings depend on the difference between WS market prices and fixed feed-in tariffs for renewable energy, whereas latter have been recently decreasing in many countries (Ibn Saif and Khadem, 2020). If this price difference is significant, prosumers are interested to sell their surplus for a higher price than the feed-in tariff and other consumers are likely to accept it, as long as it is lower than the price of their centralised supplier. Secondly, local prosumers and consumers could benefit from a reduced grid tariff for the energy supplied from local peers. Such local grid tariffs may vary between countries or regions (Radl et al., 2020) and are currently being developed in a few EU member states (Frieden et al., 2019).

LFMs aim to provide a marketplace for the DSO to procure flexibility. In case the DSO forecasts a constraint violation, it will place a corresponding flexibility order on the LFM. As a response, aggregators will bundle flexibility from local prosumers and make an offer at the LFM (Jin et al., 2020).

Another market-based instrument that is relevant – especially for the market model proposed in this paper – is Price Based Control (PBC). It is an instrument for incentivising users to adapt their load profiles according to the system’s need through price signals (Jin et al., 2020). In order to activate flexibility for the DSO, PBC can be applied in the design of network tariffs. The fixed, energy-based and power-based components of the tariff could be varied as a function of the following four dimensions (CEER, 2020a):

Time (static time-of-use): Tariff-blocks are predefined and vary for different times of the day or seasonally.
Time (dynamic): Either the change between tariff-blocks or the actual price are defined at short notice (e.g. close to real time).
Location: Different price levels depending on the users’ nodal (location in the hierarchical grid topology), zonal (e.g. zip code) or archetypical (e.g. urban/rural) characteristics. This approach is widely discussed in scientific literature as Distribution Locational Marginal Pricing (DLMP) (Sajjadi et al., 2016).
Interruptibility: Users receive an attractive tariff reduction for allowing specific devices to be interrupted during peak hours.

#### 3.2.3 Local scope

Another question arises on how the geographic extent of a local market can be defined. Kouzelis et al. (2015) addressed the geographical aspect of flexibility in distribution grids and discussed to which extent flexibility offers should be aggregated or disaggregated in an LFM. They highlight a trade-off between two basic features, which can be summarised as granularity vs. liquidity.

In an LFM, the granularity of the market and its products needs to be suitable for solving specific constraint violations (congestions, voltage violations). Hence, an aggregator wants to disaggregate its offers as much as possible in order to provide attractive flexibility services to the DSO with a high locational granularity (Kouzelis et al., 2015). Similarly, in a PBC scheme, the price differentiation between locations need to be implemented with sufficient granularity in order to be cost-reflective and to induce desired price signals. However, public acceptability for highly granular price differences between end-users might be limited (CEER, 2020a).

A well-functioning market also needs liquidity, which especially applies to P2P trading in an LEM. The more prosumers are participating, the more liquidity is reached. Hence, a rather large market area is preferred for an LEM (Mengelkamp et al., 2019). Also in LFMs, more aggregated offers are better to facilitate market processes and to minimise forecasting errors through risk diversification (Kouzelis et al., 2015).

#### 3.2.4 Market participants and market operators

The participants involved in a local market depend on the type of instrument that is implemented. In LEMs, prosumers are usually the only market participants (van Soest, 2018). A PBC framework is not an explicit market but only a market-based instrument implicitly integrated in the retail market. In the context of LFMs, the DSO is the main beneficiary procuring flexibility on the market, while the entities offering flexibility on the LFM are aggregators, bundling loads from prosumers. Direct participation of prosumers in an LFM is not considered (Torbaghan et al., 2018), because of to two reasons (Jin et al., 2020): Firstly, an individual prosumer has limited negotiating power in an LFM due to its small volume of flexibility (Burger et al., 2017). Secondly, it would overstrain the LFM in terms of communication and computation burden (Bahrami and Amini, 2018).

Apart from the market participants, the role of the MO needs to be analysed. The MO provides the trading platform and is responsible for matching offers and bids (ENTSO-E et al., 2020). In general, explicit markets (such as LEM and LFM) require the role of a MO, whereas market-based instruments that are implicitly included in the retail market (such as PBC) do not need a specific MO.

**Table 1:** Market design parameters.

Parameter	Key question	References
Purpose	What kind of energy and flexibility services should be provided?	Klaassen and Van der Laan (2019) and USEF (2015)
Market-based instruments	Which market-based instruments are applied for solving these energy and flexibility services?	Jin et al. (2020), CEER (2020b), Siano et al. (2019), CEER (2020a) and Sajjadi et al. (2016)
Local scope	How is the term ”local” defined in the context of the local market?	Kouzelis et al. (2015)
Market participants and market operator	Which roles are participating in the local market? Which entity is assuming the role of the market operator and what are its responsibilities?	Jin et al. (2020), Torbaghan et al. (2018), Repo et al. (2020), USEF (2015) and Schittekatte and Meeus (2020)
Market integration	How are the market-based instruments on local level integrated with the existing markets?	Schittekatte and Meeus (2020) and Repo et al. (2020)
Coordination of flexibility needs	Which mechanisms are introduced to prioritise the different flexibility services?	Bontius and Hodemaekers (2018) and Olivella-Rosell et al. (2018b)

In LEMs, the implementation of the MO role depends on how the balance responsibility is distributed and the settlement of imbalances is managed. Repo et al. (2020) discuss three concepts, which could be applied in P2P schemes.

1. Firstly, the MO of the LEM could act as its own BRP. In this way, the imbalance settlement mechanism would remain the same, with the BRP being fully responsible for the imbalances in its portfolio of prosumers. Hence, all prosumers engaging in the LEM would need to be part of the same balance group. This approach works as an analogue to the standard aggregator model formulated by USEF, where flexibility provision is integrated with energy supply (USEF, 2015). However, the threshold for being a BRP is quite high in terms of financial requirements and information system needs, which makes it usually not profitable to set up a BRP for local P2P trading.
2. The second option is to assign multiple suppliers to one prosumer. This means, the prosumer has several parallel contracts with different suppliers, for example one for the EV charging station, which could be used for P2P trading, and another one for the rest of the (uncontrollable) loads. This approach works as an analogue to the USEF “Virtual Transfer Points” model (USEF, 2015) and requires separate metering. Again, nothing changes regarding the traditional BRP principle.
3. Thirdly, P2P trading could be facilitated by an independent aggregator. This is an analogue to the flex-only balance responsibility model described by USEF (USEF, 2015). In this model, an aggregator that is not associated with the supplier of the prosumer is shifting loads at the prosumer’s assets and facilitates P2P trading. Therefore, the BRP associated with the aggregator needs to compensate the BRP of the supplier for the transfer of energy and any imbalances caused.

In LFMs the role of the MO can be assigned either to the DSO itself or an independent third party. The scientific controversy about this question has been analysed by Schittekatte and Meeus (2020), concluding that most arguments favour a third party MO. In order to ensure transparency and neutrality, the MO should not be a market participant simultaneously, as pointed out by Burger et al. (2019) and Ramos et al. (2016). Moreover, an engagement with a specialised third party can allow for a faster development of the procurement mechanism (Stanley et al., 2019).

#### 3.2.5 Market integration

Another important aspect for designing a local market is the question of how the new market interacts with existing markets (such as AS and WS).

In this regard, for LFMs the question arises, if they are implemented as a separate platform (e.g. where DSO can procure flexibility) or if they are integrated into other market platforms (e.g. DSO procures flexibility on the TSO’s balancing market or the WS spot market). Similar to the discussion about the MO, Schittekatte and Meeus (2020) analysed the advantages and disadvantages of having an integrated vs. separated market platforms. In integrated markets, higher liquidity can be ensured as well as complexity and costs for market participants are reduced. In contrast, the main advantage favouring a separate market platform is that the DSO can clearly specify the flexibility product according to its needs (e.g. regarding locational information).

For LEMs facilitating P2P trade, the aspect of market integration is closely tied to the question, how balance responsibility is implemented. In particular, this defines how the LEM is linked with different BRPs, who are actors on the WS market (Repo et al., 2020). For linking the LEM with the AS market, an aggregator can be considered acting as an active player in both markets (Siano et al., 2019).

#### 3.2.6 Coordination of flexibility needs

As discussed in Section 3.2.1, flexibility can be used for a variety of services requested on different markets. Therefore, it is crucial to consider coordination mechanisms prioritising the need for flexibility in order to avoid conflicts of interest among market parties. A widely discussed approach for prioritising flexibility needs is the so-called Traffic Light Concept (TLC). Especially for coordinating congestion management on DSO level, the TLC is proposed in many models (Bontius and Hodemaekers, 2018). Olivella-Rosell et al. (2018b) also apply the TLC in their local market concept and highlight that the price for flexibility should also be displayed in the TLC. For instance, in a yellow phase, when constraint violations in the distribution grid have been forecast, flexibility services for the DSO have the highest priority and consequently are also rewarded with the highest remuneration.

All the market design parameters reviewed in this section are summarised in Table 1 including the key question they address and selected literature references where they have been discussed.

## 4 Barriers hindering the adoption of Local Energy and Flexibility Markets

### 4.1 Identification and prioritisation of barriers

Drawing on the literature about the proliferation of energy-efficient or clean technologies it was found that main barriers are usually more immaterial than physical (e.g. understanding of pricing structures, comprehension of political and regulatory frameworks or disillusion administrative processes or the benefits that the new technology entails). Although a large number of publications have been published in the body of literature discussing the obstacles to the adoption of these technologies (e.g. renewables (Seetharaman et al., 2019, Asante et al., 2020), demand response (Good et al., 2017) or DERs (Spiess and De Sousa, 2016)) to the best of the authors’ knowledge, there were not prior research studies exploring in detail the degree in which each barrier may affect the adoption of LFMs or LEMs. While the authors of this manuscript identified them in a previous publication (Zabaleta et al., 2020), this section, apart from reviewing the main findings, quantifies their effects on the design of new market models and the actors that have to cope with them.

#### 4.1.1 Identifying and classifying barriers — taxonomy

It was identified that the most common barriers that hinder the LFM proliferation include: the lack of previous experience of energy companies, the use of Blockchain technology for verifying transactions, the initial investment that companies and prosumers have to make, lack of regulations in most countries, the complexity of the systems and contracts to be understood by prosumers and finally personal data and privacy concerns.

**Fig. 2:**
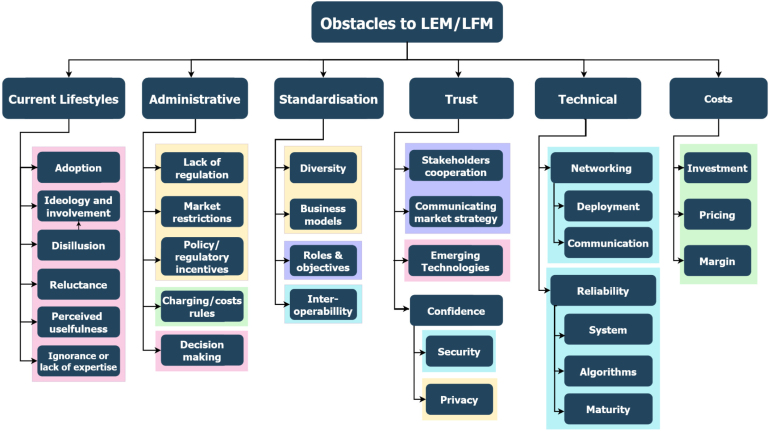
Taxonomy of the barriers identified for the adoption of Local Flexibility Markets (Zabaleta et al., 2020). The different colours of the subcategories reflect: pink (social barriers), yellow (regulatory barriers), green (economic barriers), turquoise (technical) and purple (a mix of different barriers).

Each of these barriers encountered through a triangulation method (review of the literature, interviews with experts and surveys with end-users) were clustered in categories and subcategories as can be observed in Fig. 2. As the complexity of the taxonomy is high, each of the subcategories were thoroughly explained in the previous publication (Zabaleta et al., 2020), but the main findings are provided in Appendix of this manuscript. From left to right of the Figure, the categories reflect the number of times they were identified in the triangulation method. Hence, current lifestyles was the most recurrent barrier and cost the least identified.

#### 4.1.2 Prioritising barriers - Delphi method

The next step was to assess the relative importance of each barrier category for the energy market actors. To this end, the researchers decided to employ the Delphi method (Linstone et al., 1975) as the team have conducted several research actions applying it. This approach is an iterative procedure designed to help a panel of experts to reach consensus about a topic. For the endeavour of this paper, fourteen market actors from the PARITY project were recruited, namely: one facility manager, six researchers on energy markets, one business innovation consultant, one project manager in the energy sector, one developer of services in an Energy Service Company (ESCO), one CEO of a DSO, one DSO’s project manager, one Chief Operating Officer (COO) of a smart grid startup and one aggregator. The Delphi was conducted by one researcher with two other researchers with the support role in each session. The task that each expert had to perform in each session was to score, according to their understanding, the relevance of each barrier’s category from −5 (not relevant at all) to 5 (extremely relevant) for every energy market roles obtained from the USEF role model (Klaassen and Van der Laan, 2019). In the second and the third round their scoring results were presented as average measures. The goal of these latter sessions was to openly discuss the average agreements and disagreements to reach a consensus.

As can be observed in Table 2, each of the first-tier barriers were linked to the participant roles in the PARITY ecosystem. The main conclusions obtained were that the list of barriers that can impact more on the proposed use-cases in the PARITY project, were those related to standardisation (using the critical set and average scores). Moreover, trust and costs were also highly relevant for the project when it comes to designing the overall architecture. A very important finding was observing that current lifestyle (which was the most prominent barrier quantitatively and one of the most cited barriers in the body of knowledge) was, according to experts, only affecting prosumers and no other actors. Therefore, the analysis revealed that this barrier has to be treated in isolation from the others with mixed-methods to explore how to overcome the lack of understanding of LFMs and LEMs or the low perception of it usefulness in future energy distribution and management.

**Table 2:** First-tier barriers against actors in LFM/LEM after three rounds of Delphi method.

Roles	Lifestyles	Administration	Technical	Trust	Costs	Standardisation
Prosumer	X			X	X	
BRP		X		X		X
Supplier		X		X	X	X
DSO		X	X	X	X	X
TSO			X			X

### 4.2 Conflicts of interest

After concluding the Delphi method and prioritising the barriers, it was noticed that conflicts of interest may arise between the different market roles mentioned in Table 2. These conflicts are described hereafter.

DSO – Supplier: Profits optimisation between sold and acquired energy: While the DSO is responsible for maintaining the distribution grid and avoiding congestion, the supplier needs to optimise the profit between sold and acquired energy. Thus, conflicts may arise, considering that price signals (e.g. in a Time-of-use pricing scheme) or demand response interventions coming from the electricity supplier do not reflect the grid status and therefore might create congestion in the distribution grid.
DSO – Supplier: Reliable data exchange increases DSO costs: The collection and processing of data is a responsibility of DSOs. Although in local market models, data processing will be required in higher time resolution. This will place pressure on the processes of DSOs, while suppliers may benefit from this pattern.
Prosumer - DSO: Prosumers privacy: Meter mechanisms collect information on prosumers activities. The prosumer though, does not know who owns the information, who can control it, and who can profit from it. Controlling data entails a slew of duties for the DSO. Prosumers’ privacy must be protected at any stage.
Prosumer - Supplier: The liability is transferred to the prosumers through dynamic tariffs: Dynamic tariffs are gaining momentum. The probability of price uncertainty is transferred to the prosumers and unwelcome surprises in energy bills can occur.
DSO - TSO: An interface for data communication is required: The advent of DERs necessitates coordination between DSO and TSO in order to maintain grid stability. Building an interface is complex, and these players’ perspectives on how it can be done differ.

The above conflicts of interest hinder future shifts in roles and obligations from the viewpoints of various stakeholders. The proposed market model presented in the following section will attempt to address these issues.

## 5 Proposed hybrid market model of PARITY

As previously identified, it is a main barrier that end-users have difficulties to understand the concepts of LEMs and LFMs and might be reluctant to participate in such markets as a result. Hence, the authors propose to apply a hybrid market, where both an LEM and an LFM are implemented, but the end-users are exposed only to one single transparent price signal.

### 5.1 Purpose of the PARITY market framework

The aim of the local market in PARITY is threefold. Firstly, the overarching purpose is to provide flexibility services for the DSO to perform congestion management and voltage control. This should be enabled in an efficient way, creating a level-playing field for different kinds of competitive flexibility providers.

Latter implies, secondly, that especially small-sized prosumers connected to the distribution grid should be able to participate in the flexibility provision. On the one hand, this is because constraint violations in the distribution grid can be only solved with flexibility from local assets and on the other hand, it is intended to empower prosumers to participate in various electricity markets. This also means, local prosumers should be granted access to existing AS/WS markets as well.

Thirdly, flexibility should be activated in a transactive way, meaning through energy transactions among prosumers. In order to achieve this, the transactions need to be steered by price signals, enabling prosumers to react on them and activate the desired flexibility as a result. Hence, explicit flexibility requests need to be translated into price signals taking into account the price elasticity of heterogeneous prosumers in the distribution grid.

Finally, the proposed model should be applicable in diverse regulatory frameworks that might emerge in the future among EU member states. This refers to the way DSOs are entitled to meet their flexibility needs.  [9] mentions (a) rules-based flexibility requirements, (b) network tariffs, (c) connection agreements and (d) market-based procurement as possible options to be determined by the national regulatory bodies. For the market model in this paper, market-based procurement and adapted network tariffs are considered.

### 5.2 Market-based instruments applied in PARITY

In order to fulfil the purpose, the proposed hybrid market model consists of two novel markets: An LEM and an LFM.

On the LEM, prosumers can trade electricity with each other in real time. This is facilitated by a fully automated and smart contract-based LEM platform, operated by a novel role, the so-called Local Energy Market Operator (LEMO). The main benefits of the LEM are cost savings for the prosumers, which arise from the difference between wholesale prices and feed-in tariffs as well as from reduced grid tariffs on local level. Additionally, the LEM in the PARITY market model serves as a means to activate flexibility from the prosumers. However, there are certain limitations for this: Firstly, P2P trading as such does not influence physical power flows, but rather the allocation of costs. Therefore, flexibility benefits can only be achieved, if prosumers can react to local market prices and adapt their consumption profile accordingly. Secondly, P2P trading on an LEM could indeed lead to self-balancing of a local community of prosumers, but could also lead to new problems in the grid. For instance, if a few prosumers with flexible loads purchase a high amount of surplus electricity from local peers in a short time frame, this could lead to congestions at lower level nodes. This means, an LEM as a standalone instrument does not contribute to avoiding congestions or voltage violations in the grid.

To solve this, also an LFM is implemented in the proposed market model. As the DSOs’ activities are part of the regulated domain, the design of this instrument is up to regulatory bodies. In this paper, two alternative concepts are considered: the explicit and the implicit LFM.

The explicit LFM represents a standalone market platform where the DSO procures flexibility in a market-based way. For operating this marketplace, another novel role is defined, the so-called Local Flexibility Market Operator (LFMO). The DSO forecasts potential constraint violations in its grid area and places a request on the LFM platform. Here, flexibility products are considered unconditional, meaning that there are no long-term reservation payments for keeping available flexibility resources. This is because the proposed interaction with real time energy trading on the LEM would be hampered by capacity reservation, as LEM participants might not be able to guarantee availability of their flexible assets on a long-time horizon.

As an alternative option, in the implicit LFM the DSO applies a novel network tariff. In this way, the activation of flexibility is implicitly achieved in the LEM and there is no market platform for the LFM. The implicit LFM is designed as a PBC mechanism. The DSO is forecasting potential constraint violations and the location of the respective congestion points and determines locationally differentiated grid prices. If the prosumers react to these price signals by altering their load and/or generation profiles, constraint violations can be avoided indirectly. The alteration of the load profile goes along with changed trading behaviour on the LEM. For example, a consumer facing a significant increase of the grid price will decrease the load and as a result will purchase less energy from the peers. Meanwhile, another prosumer may face a reduction in the grid price and will try to increase its load by charging its EV, for instance. From a regulatory perspective, in this scenario the national regulator needs to allow spatio-temporally varying grid tariffs.

Finally, the link between the LEM and the explicit or implicit LFM needs to be defined. This link is a core element of the market model proposed in this paper and describes, how flexibility can be activated through energy transactions among prosumers. The idea of this mechanism is to convert all flexibility needs of the DSO into price signals, irrespective of the regulatory decision to either enable market-based flexibility procurement or adapted network tariffs. In both scenarios price signals are communicated to building energy management systems or smart home gateways that can react accordingly.

**Fig. 3:**
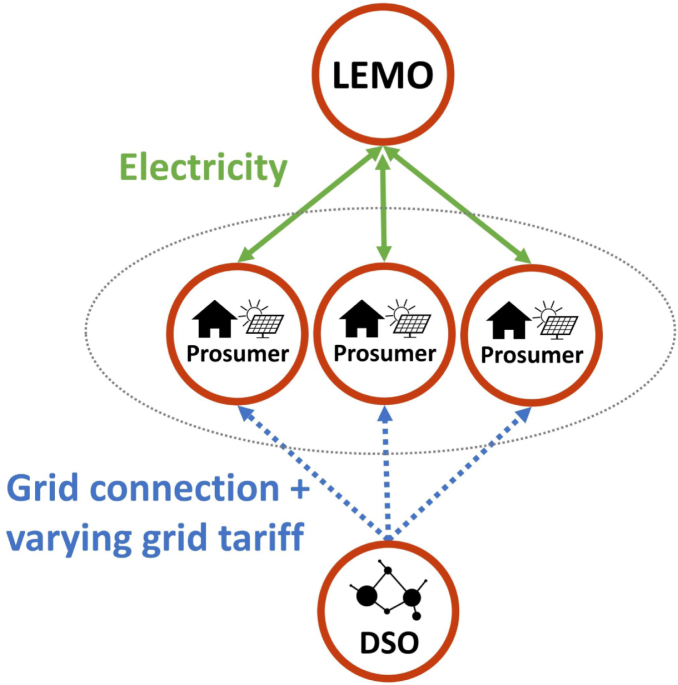
Schematic of the LEM with an implicit LFM.

In case an implicit LFM is implemented, the DSO imposes these price signals directly on the prosumers as a part of the grid tariff. In this scenario, the LEMO is not aiming at activating flexibility for the DSO, but just facilitates P2P trading. The prices on the LEM platform only reflect demand and supply between local prosumers. However, the transactions are also indirectly affected by the price signals coming from the DSO, because they need to be added to each P2P trade and paid by the prosumers to the DSO as a grid tariff. The schematic of the interconnection between LEM and implicit LFM is shown in Fig. 3.

**Fig. 4:**
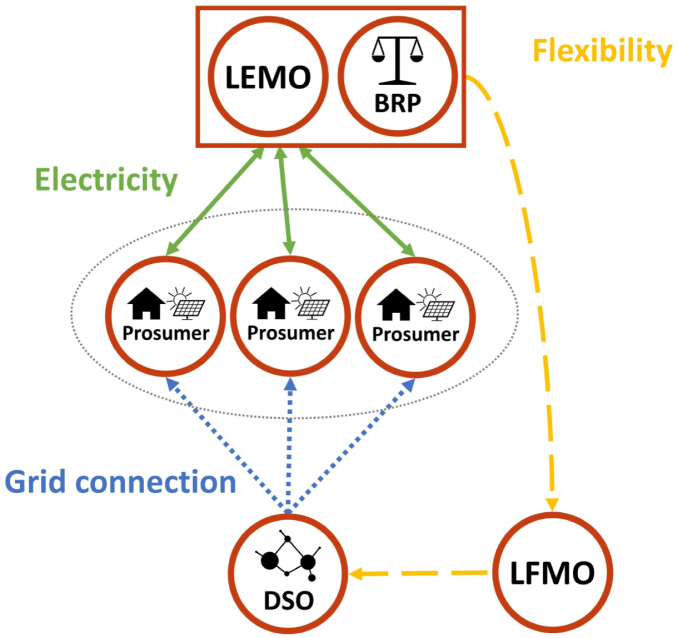
Schematic of the LEM with an explicit LFM.

If the LFM is designed as an explicit marketplace, the flexibility requests of the DSO need to be translated into price signals for the prosumers. Firstly, the LEMO sells flexibility (via a BRP) to the DSO on the LFM, just like an aggregator. The price for the flexibility bundle is determined through the clearing on the explicit LFM. Then the price signals for the individual prosumers involved in this flexibility bundle are determined by the LEMO, who needs to make sure, that the agreed flexibility is actually activated. Therefore, the LEMO charges a spatio-temporally varying fee on each LEM trade, reflecting the remuneration gained from the flexibility provision on the LFM. This means, for prosumers who should ramp down consumption in order to solve the grid problem this fee will be high, whereas for prosumers who should ramp up their consumption this fee will be low or even negative. The key challenge for the LEMO in this context is to find the right price signals to actually steer the prosumers’ behaviour. To make the price signals as accurate as possible, the LEMO needs to carefully consider the price elasticity of the prosumers in the LEM, which is mainly determined by the available DERs and their flexibility potential. However, compared to the DSO, the LEMO might be in a better position to assess the price elasticity, which is because of its insight in LEM trades. Also, the LEMO is not a regulated entity and therefore has more freedom for designing its pricing scheme.^1^ This could be beneficial in order to achieve high local granularity of the price signals and precisely avoid constraint violations in the grid. The schematic of the interconnection between LEM and explicit LFM is shown in Fig. 4.

### 5.3 Definition of the local scope

Constraint violations occur at physical locations in the distribution grid. Hence, the definition of the local scope should be “nodal”, meaning derived from the grid’s hierarchical topology. CEER (2020b) highlights two main technical prerequisites enabling DSOs to avoid constraint violations. The first one, observability, refers to the DSO’s ability to assess the state of the network and identify possible problems. The second one, controllability, means the DSO’s ability to steer flexible assets in the grid, which can be done directly or through indirect measures. For defining the local scope of this market model, within the Delphi process representatives of DSOs from three different EU member states have discussed their current level of observability and the their needs for controllability.

**Fig. 5:**
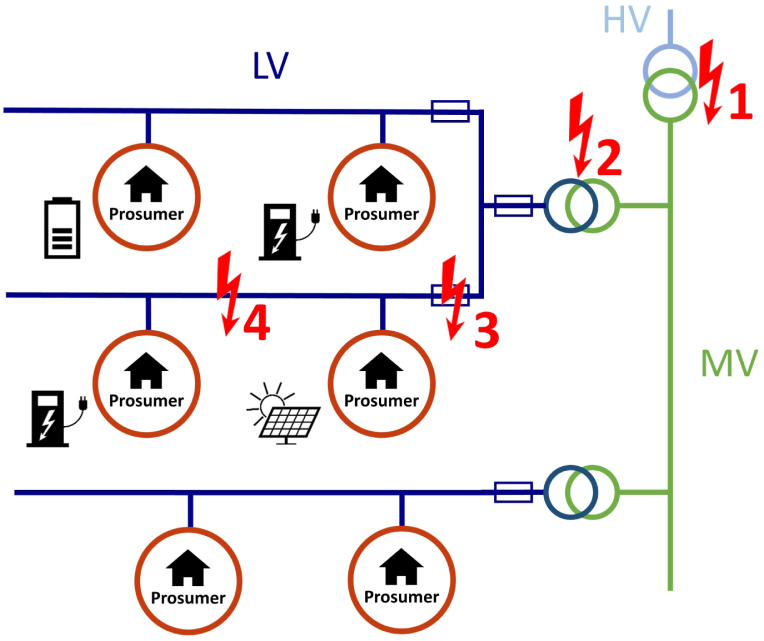
Typical locations of constraint violations in distribution grids.

Regarding observability, all experts agreed that DSOs can mainly detect constraint violations through direct measurement of current and voltage at transformer stations, feeders and increasingly also prosumers’ smart meters. Regarding controllability, control of the load flows would be desired at following locations in the grid topology, where constraint violations may occur (Fig. 5):

1. At High Voltage (HV)/Medium Voltage (MV) transformer stations
2. At MV/Low Voltage (LV) transformer stations
3. At LV feeders from MV/LV transformer stations
4. At power lines between prosumers.

Finally, the DSO representatives concluded that constraint violations at power lines between prosumers (type 4) might be the most critical ones in the future. This is mainly because of two reasons: Firstly, type 1–3 constraint violations are currently the most typical problems in distribution systems, which is also because of the high level of observability at these points. Therefore, there are already procedures in place to tackle these problems, e.g. by changing the tap configuration at a transformer station. However, type 1–3 are still relevant problems to be considered when designing an LFM. But in contrast, secondly, type 4 constraint violations are expected to rapidly increase in the future due to electricity feed-in from fluctuating Photovoltaics (PV) generation and new loads such as heat pumps and EVs, which are decentralised and can be newly installed at any location of the grid, putting pressure on existing power lines. With smart meter roll out gaining pace, observability increases for these type of problems, but there is still a lack of mechanisms how the DSOs can steer load flows to avoid them.

**Table 3:** Summary of the PARITY market model — decision tree and activities per role.

Decision	DSO traffic light	GREEN	YELLOW		RED
	National regulation		Explicit LFM	Implicit LFM	
Activities	DSO	Normal grid tariffs	Normal grid tariffs + flexibility procurement	Spatio-temporally varying grid tariff	Direct load control
	LEMO	C&S of P2P trades + offer flexibility to Aggregator + adapt overall price levels	C&S of P2P trades + offer flexibility to DSO + adapt individual price levels	C&S of P2P trades (+ consider DSO tariffs for load shifting)	–
	Aggregator	Request flexibility from LEMO	–	–	–
	LFMO	–	C&S of explicit LFM	–	–
	Prosumer	Automatically trade electricity P2P, adapting to prices from			–
		LEMO	LEMO	LEMO and DSO	–

C&S means clearing and settlement.

As a result, the PARITY market model should not only tackle type 1–3 problems, but also the emerging type 4 problem. Hence, the authors propose to include the whole area of a distribution grid in the local scope of the hybrid market model. On the one hand, this safeguards the liquidity on the LEM, with the P2P trades running on the blockchain. On the other hand, the local granularity that is necessary for the LFM can be dynamically adjusted, through spatio-temporally varying grid prices from the DSO (implicit LFM) or price signals from the LEMO (explicit LFM). Keeping the local granularity of the LFM dynamic is also an efficient approach in terms of computational burden, as prices only need to be recalculated in the area affected by the constraint violation (e.g. the area of a LV feeder), but not the whole distribution grid.

### 5.4 Market participants and market operators in PARITY

On the LEM proposed in this hybrid market model, prosumers are the only direct market participants, trading energy P2P. On the explicit LFM, the DSO is the only consumer of flexibility, whereas the participants making flexibility offers are the LEMOs, who act as intermediaries between prosumers and the explicit LFM. In line with the literature discussed above, prosumers are not included as direct actors in the explicit LFM. Also, BRPs are not considered as participants in the LFM, because they are participants of the WS market and do not need to meet their flexibility demand by procuring local flexibility (Ibn Saif and Khadem, 2020). Note, that BRPs are still affected by the local market activities, as the transfer of energy might have an impact on their balances.

The LEMO is responsible for providing the technical infrastructure, enabling the prosumers to offer and make bids on the LEM, fully automated and based on smart contracts. Also, the LEM is cleared (matching offers and bids) and settled (invoicing the traded energy) by the LEMO. In general, the role of the LEMO is assumed by a private competitive entity. This is fully in line with unbundling principles, which require regulated entities like the DSO not to be involved win energy supply (where P2P supply is part of) and minimise their contact to the end-users other than a DSO, a private LEMO does not have a natural monopoly. It acts as a P2P facilitator, which is a competitive role like a supplier. Indeed, the LEMO could be in a dominant position as the only LEMO available. Therefore, it is important to notice, that end-users are not obliged to be part of the LEM; it is an optional service. Similarly to the open participation in an energy community, end-users can choose if they want to join an LEM or even opt for a competing LEM in the same area.

Considering balance responsibility for P2P trading (Section 3.2.4), in this paper either an energy supplier or an energy community is considered for the role of the LEMO. If the LEMO is a supplier, all the P2P trades would be conducted within the same balance group, keeping the balance position of the BRP unchanged. In this case, all of the prosumers in the LEM need to have a supply contract with the LEMO for each (sub)metering point. Having an energy community as the LEMO, this would be in line with the emerging concepts of Citizen Energy Community (CEC) (EU, 2019) and Renewable Energy Community (REC) (EU, 2018), where latter have a clear local focus. The energy community administration can assume the role of the LEMO by acting as an independent aggregator, which is dissociated from the supplier role. In this way the free choice of supplier is guaranteed within the energy community.^2^

In the explicit LFM, the LFMO is responsible for providing, administrating, clearing and settling the LFM platform. In this market model, the role of the LFMO is assumed by a regulated entity, that needs an authorisation by the national regulator. This could either be the DSO itself or another regulated entity (e.g. MO of another organised market). As this is a regulatory decision, the PARITY market model is agnostic about that and aims to be compatible with different types of explicit and implicit LFMs.

### 5.5 Market integration with AS/WS markets

As this market model also aims at giving prosumers access to AS and WS markets, the interaction of the novel local market with existing markets needs to be defined.

**Fig. 6:**
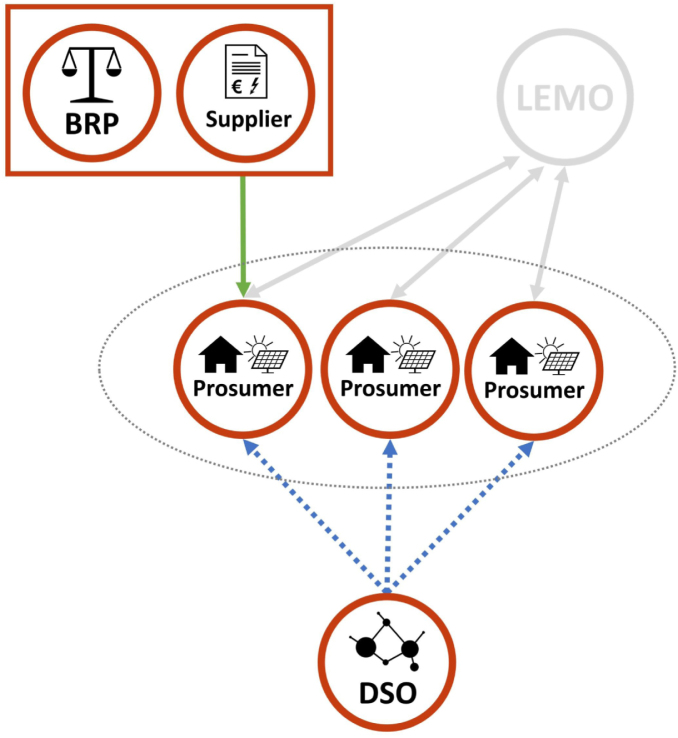
Schematic of linking LEM with WS market via supplier.

The most straightforward link between the prosumer and the WS market is established through the energy supplier. The prices on the LEM are influenced by the pricing scheme of each prosumer’s supplier (e.g. time-of-use or real time pricing) and therefore by the prices on the WS market (Fig. 6).

**Fig. 7:**
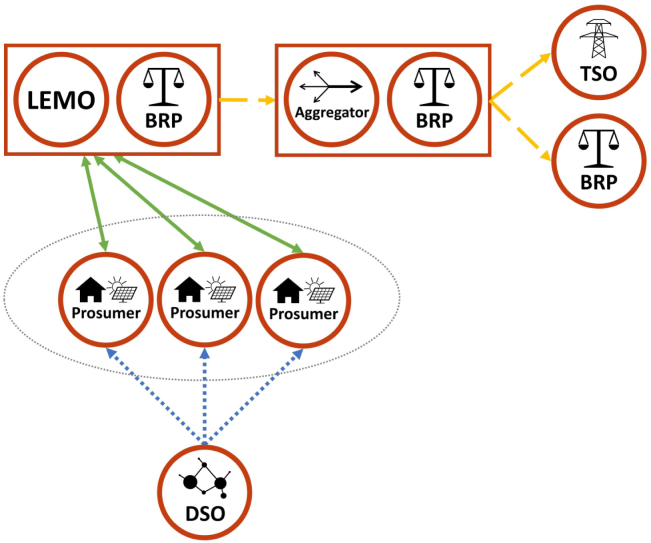
Schematic of linking LEM with AS/WS markets via aggregator.

Additionally, the role providing the link from the prosumers to the AS/WS markets is fulfilled by an aggregator via the associated BRPs (Fig. 7). The PARITY market model proposes to convert explicit flexibility requests from these markets into price signals that can be passed on to the prosumers. This means, the LEMO offers explicit flexibility to an aggregator who is trading on the AS/WS markets. To activate this flexibility, the LEMO adapts the overall price levels in the LEM accordingly to steer the prosumers’ behaviour. In order to reach an effective signal when setting these price levels, the LEMO needs to consider the price elasticity of the whole pool of prosumers in the LEM.

However, the risk for actually delivering the explicit flexibility remains with the LEMO. Hence, the uncertainty of the prosumers’ reaction to specific price signals needs to be minimised through proper forecasting and automated dispatching. This risk is most critical in the balancing market, where also reservation mechanisms apply, making uniform price signals (for reservation and dispatch) not feasible. Hence, a LEMO can only offer its flexibility to an aggregator with a sufficiently large portfolio, where there are stochastic redundancies in case some flexibility providers are not able to deliver as expected. As long as there is a high diversification in the aggregator’s portfolio in terms of asset types and locations, LEMOs could participate in such a pool also for AS market offers, if national regulation allows.

### 5.6 Coordination of flexibility needs — the PARITY traffic light approach

To prioritise flexibility needs, in this market model the application of a TLC is proposed. The formulated TLC differentiates three grid operation regimes, which are determined by the DSO and reflect the state of the distribution grid according to the DSO’s forecast.

Firstly, in the GREEN grid operation regime, no constraint violations are expected. Hence, prosumers can freely perform P2P trading and also aggregators can request flexibility from the LEMO. The YELLOW grid operation regime is determined in case some constraint violations are forecasted. Solving these problems efficiently, the DSO procures flexibility on the explicit LFM or enforces the spatio-temporally varying grid tariff. To give priority to the DSO’s needs, during this regime the LEMO stops to fulfil further flexibility requests from the aggregator. However, P2P trading in the LEM continues, but with an additional price signal either from the DSO (implicit LFM) or the LEMO (explicit LFM). Note, that the transition between the GREEN and YELLOW regime is seamless as the limitation of aggregator requests and the additional DSO measures can be enforced incrementally, depending on the actual needs at affected network nodes. In case the market-based activities to obtain flexibility for the DSO do not suffice, the RED grid regime is determined. In this scenario, all market-based activities are paused (including P2P trading) and the DSO takes over control by disconnecting specific loads that endanger grid stability.

The whole PARITY market model proposed in this paper is summarised in Table 3. It shows the decision parameters (grid regime and regulation on grid tariffs) and accordingly the activities of each market role in the different scenarios.

## 6 Numerical examples

In order to illustrate the monetary benefits that could be gained by consumers and prosumers, the hybrid market model of PARITY has been applied in a set of numerical examples. These examples serve as a preliminary validation and indicate the order of magnitude of the expected financial implications.

### 6.1 Example specification

For the examples presented in this section, a simplified local market with three participants A, B and C is considered. For modelling their load profiles, a standard household profile $H_{0}$ is applied, depicting an average weekday during shoulder season (April) in Austria. Each profile is scaled up to an annual energy consumption of 5000 kWh (e.g. single family house). Participant A represents a prosumer with an 8 kW${}_{p}$ PV installation (total generation of 8000 kWh per year), whereas B and C are pure consumers. It is assumed that each participant has a flexibility potential of 20%, meaning that at each time of the day they can ramp up or down their profile by 20% of the current load.

The price information is retrieved as an example also from Austria. An energy supply price for centrally sourced renewable energy of 0,08 /kWh is assumed. Network tariffs and taxes amount to 0,12 /kWh and are reduced by 60% for the energy traded locally in the LEM, resulting in reduced tariff and taxes of 0,048 /kWh (Radl et al., 2020). The energy prices of the energy traded locally in the LEM result from an inverse function of the generation profile of the PV plant of participant A. The minimum energy price (at time of maximum PV surplus) is 0,04 /kWh, which is currently a typical market-based feed-in offering in Austria and corresponds to the overall average day-ahead price in 2019 (ENTSO-E, 2019). The maximum energy price is 0,152 /kWh, which is equal to the competing supplier price considering the different network tariff and taxes (Table 4).

In order to adapt the price signals on the local market, a simplified iterative mechanism has been applied. Note, that this is not the final mechanism eventually applied in the PARITY pilots but only serves for the preliminary validation in the numerical examples. In case negative flexibility is demanded (due to excess energy in the grid), the prices in that period are reduced by 0,001 /kWh per iteration to trigger increased consumption. Vice versa, in case positive flexibility is demanded (due to energy deficit in the grid), the prices in that period are increased by 0,001 /kWh, but the prices in every other time of the day are reduced by 0,001 /kWh. This mechanism should trigger flexibility during the time period where it is required and at the same time ensure positive profits for the prosumers and consumers. To simulate optimal load shifting behaviour as a reaction to these price signals, an optimisation problem has been formulated, minimising the overall costs with the objective function

$$\min\overset{T}{\underset{t=0}{\sum }}C\left(L_{O}\right),$$

where

$$C\left(L_{O}\right)=L_{O}\left(t\right)\cdot s_{LEM}\left(t\right)\cdot \left(p_{LEM}\left(t\right)+\alpha \left(t\right)\right)++L_{O}\left(t\right)\cdot s_{S}\left(t\right)\cdot \left(p_{S}+\alpha \left(t\right)\right),$$

$$s_{LEM}\left(t\right)=\min\left\{\begin{array}{c} G_{surp}\left(t\right)∕L_{O}\left(t\right)\;\\ 1,\; \end{array}\right)$$

$$s_{S}\left(t\right)=\max\left\{\begin{array}{c} \left(L_{O}\left(t\right)-G_{surp}\left(t\right)\right)∕L_{O}\left(t\right)\;\\ 0,\; \end{array}\right)$$

and C is the total cost for all market participants A, B and C in , $L_{O}$ is the optimised load profile in kWh per 15 min interval for all market participants, $s_{LEM}$ and $s_{S}$ are the shares of energy obtained from the LEM and the centralised supplier respectively, $p_{LEM}$ and $p_{S}$ are the prices in  on the LEM and of the supplier and α is the price signal in  introduced above. $G_{surp}$ represents the surplus generation of prosumer A, which is available for being traded on the LEM.

**Table 4:** Pricing and tariff information.

	Energy price [/kWh]	Network tariff and taxes [/kWh]
Supplier price	0,080	0,120
Maximum LEM price	0,152	0,048
Minimum LEM price	0,040	0,048

The objective function is subjected to following three constraints:

(1) $$L_{O}\left(t\right)\leq L_{B}\left(t\right)\cdot \left(1+f\right),$$

(2) $$L_{O}\left(t\right)\geq L_{B}\left(t\right)\cdot \left(1-f\right),$$

(3) $$\overset{T}{\underset{t=0}{\sum }}L_{O}\left(t\right)=\overset{T}{\underset{t=0}{\sum }}L_{B}\left(t\right),$$

where $L_{B}$ is the total base energy consumption profile of A, B and C without any load shifting and f is the share of shiftable loads, which is considered a constant of 20%, as mentioned above. Constraint (1), (2) ensure that the optimised load profile $L_{O}$ is not altered by more than ±20%, meaning that the available flexibility potential of the market participants is not exceeded. Constraint (3) defines that the amount of energy consumed remains the same in the optimised profile and only load shifting is induced by the price signals. Note that under normal conditions, a flexibility action includes an overall increase in the energy consumption due to a loss of efficiency. However, in order to keep the model simpler, this effect has been omitted in these examples.

For prosumer A, the energy consumption profile already considers self-consumption optimisation. This is the first priority of prosumer A, as no fees and taxes apply for the electricity consumed on-site. Hence, prosumer A will use its flexibility potential entirely for self-consumption optimisation and then offer the surplus generation on the LEM and in this way further reduce its costs.

### 6.2 Example results

The optimisation problem is solved by a Generalised Reduced Gradient (GRG) nonlinear solver for one day. The algorithm optimises the load profile based on the price profile in different scenarios.

Scenario 0 represents the Business as Usual (BaU) case with no LEM deployed. Scenarios 1 and 2 consider an LEM, but both with fully inflexible demand, so still without any load shifting through the optimisation algorithm. Whereas Scenario 1 considers a reduced grid tariff for locally generated energy (such as in a REC), Scenario 2 considers the normal tariff for all the energy consumed (such as in a CEC).

Then, in Scenarios 3 and 4 the loads are considered flexible and are optimised by the optimisation algorithm stated above. The price profile used for the optimisation considers the prices for locally traded energy on the LEM, again with and without a reduced grid tariff respectively.

The optimisation in Scenario 5 also includes requests from aggregators who are active on the balancing market. Requests for negative flexibility in a time slice between 04:00 and 08:00 a.m. and positive flexibility between 08:00 and 12:00 p.m. are considered, resulting in an adapted price profile according to the pricing mechanism described above. Scenario 6 also considers the same aggregator requests, but the remuneration is not determined by the iterative pricing mechanism, but by the average actual market price on the balancing market. Finally, Scenario 7 represents a YELLOW grid regime (hence an LFM scenario), where a local feeder is congested and the peak load of the local market needs to be reduced. Again, the iterative pricing mechanism is applied to incentivise load shifting during this peak.

Table 5 gives an overview of the scenarios including the active markets and the number of price iterations that have been applied to trigger the desired optimisation.

The results of the costs in each of these scenarios are summarised in Table 6 below. At first, the total costs per market participant are compared. Then the cost savings in each scenario compared to the BaU (Scenario 0) are presented both in absolute values [] and as a ratio [%]. The right part of the table shows for each scenario the load that has been shifted in total (up or down) and during the requested time period. Note that negative flexibility is indicated by negative values, but refers to increasing consumption and vice versa. Also, the total payment of the DSO or aggregator towards the flexibility providing market participants are shown as well as the resulting price per kWh of shifted energy consumption.

**Table 5:** Scenario definition.

Scenario		Local grid tariff	Load shifting	Grid regime	LEM	LFM	AS/WS market	Iterations
0	BaU	No	No	G	–	–	–	0
1	LEM	Yes	No	G	Yes	No	No	0
2	LEM no reduced tariff	No	No	G	Yes	No	No	0
3	LEM + load shift	Yes	Yes	G	Yes	No	No	1
4	LEM + shift no reduced tariff	No	Yes	G	Yes	No	No	1
5	LEM + AGG	Yes	Yes	G	Yes	No	Yes	1
6	LEM + AGG	Yes	Yes	G	Yes	No	Yes	–
7	LEM + LFM	Yes	Yes	Y	Yes	Yes	No	2

**Table 6:** Scenario results.

Scenario		Cost []				Cost savings []				Cost savings [%]				Load shifted [kWh]	Flexibility activated during requested period [kWh]		DSO/ Aggregator payment []	Flexibility price per kWh []
		A	B	C	Sum	A	B	C	Sum	A	B	C	Total		Neg.	Pos.		
0	BaU	0,40	2,79	2,79	5,99	–	–	–	–	–	–	–	–	–	–	–	–	–
1	LEM	0,06	2,35	2,35	4,76	0,34	0,45	0,45	1,23	84	16	16	15	–	–	–	–	–
2	LEM no reduced tariff	0,28	2,63	2,63	5,55	0,12	0,16	0,16	0,44	30	6	6	5	–	–	–	–	–
3	LEM + load shift	0,04	2,27	2,27	4,57	0,36	0,53	0,53	1,41	90	19	19	17	1,66	–	–	–	–
4	LEM + shift no reduced tariff	0,27	2,60	2,60	5,48	0,13	0,19	0,19	0,51	32	7	7	6	1,66	–	–	–	–
5	LEM + AGG	0,04	2,26	2,26	4,55	0,36	0,54	0,54	1,44	91	19	19	17	2,42	−0,66	1,16	−0,02	0,0128
6	LEM + AGG market price	0,04	2,21	2,21	4,46	0,36	0,58	0,58	1,52	91	21	21	19	2,42	−0,66	1,16	−0,11	0,0592
7	LEM + LFM	0,03	2,24	2,24	4,52	0,37	0,55	0,55	1,47	92	20	20	18	2,55	–	1,03	−0,05	0,0510

**Fig. 8:**
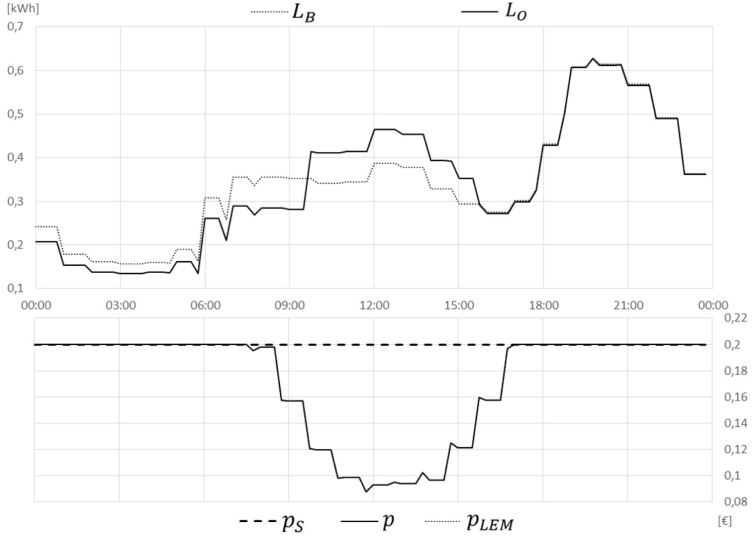
Scenario 3 LEM + shift.

In the BaU Scenario, the total cost sums up to 5,99 for all market participants in the observed day. Only 0,40 apply to prosumer A, as selling the surplus for a fixed feed-in offering of 0,04/kWh reduces its bill. The LEM introduced in Scenarios 1 and 2 significantly reduce the overall costs. Especially in the case of reduced grid tariffs (Scenario 2), the costs are reduced by 15% in general and even by 84% for the prosumer A who can sell its surplus for a higher price to local peers. Introducing load shifting for optimally making use of the LEM prices in Scenarios 3 and 4 further leads to a small cost reduction, with 17% in Scenario 3 compared to BaU. This is shown in Fig. 8 indicating in the upper part the BaU profile $L_{B}$ and the optimised profile $L_{O}$ and in the lower part the final adapted price level p, the supplier price $p_{S}$ and the price on the LEM $p_{LEM}$.

**Fig. 9:**
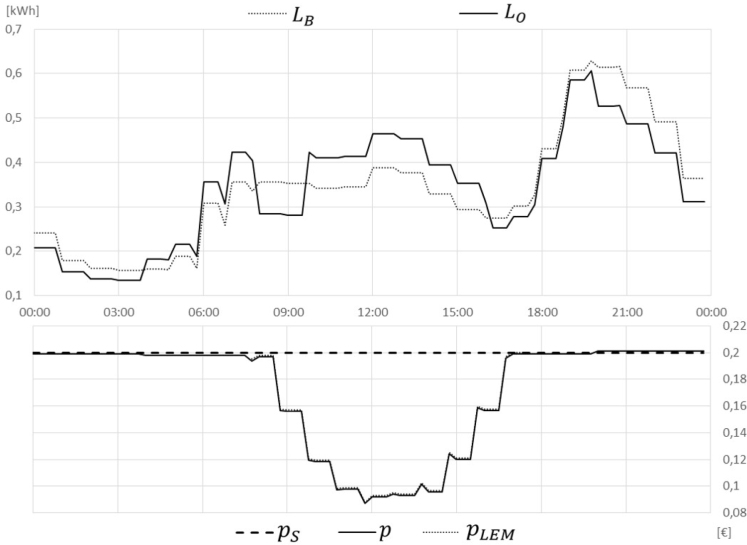
Scenario 5 LEM + AGG.

Including also aggregator requests (Fig. 9) only has a minor impact on the cost savings. This is especially negligible if the minimum prices that lead to a behaviour change are applied (Scenario 5), which result from the iterative price adaption process. In case the actual balancing market prices are applied (Scenario 6), the savings increase but are still small. The chosen market price in Scenario 6 is approximately 0,06/kWh which is the average price for activated balancing energy on the aFRR+ market in Austria in the year 2020 (ENTSO-E, 2020).

**Fig. 10:**
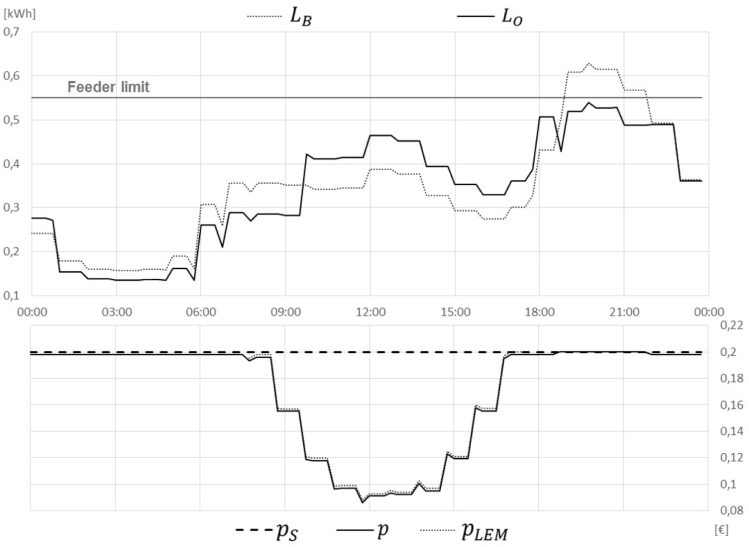
Scenario 7 LEM + LFM .

Another small cost reduction is achieved by introducing the LFM in YELLOW grid regime (Fig. 10), instead of the aggregator requests. The feeder simulated in this example shows a maximum feeder capacity of 0,55 kWh per 15 min time slice (which is equal to 2,2 kW), leading to a congestion during evening peak. In order to not violate this constraint, two iterations of the optimisation algorithm needed to be executed, resulting in a flexibility price of about 0,05/kWh. However, as the time period of such a peak is usually rather short, the resulting cost savings are also rather small with total savings of 18% compared to BaU. Note, that this feeder limit is a very small theoretic value as in practice one feeder does not serve only such a small community.

Concluding these numeric example, it can be argued that most of the cost savings result from the general idea of P2P trading in an LEM and also the reduced grid tariffs that apply in such a scenario. Compared to that, the gains through load shifting in several respects (for optimally using LEM prices, serving aggregator requests or reacting to DSO constraints in an LFM) are expected to be rather small.

## 7 Discussion and implications

**Fig. 11:**
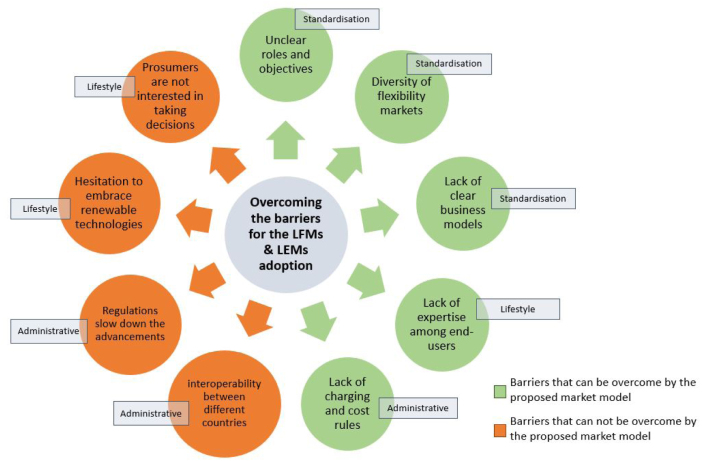
Barriers that can be and cannot be overcome by the proposed market model.

This section provides a brief discussion on key aspects regarding the strengths and weaknesses of the proposed hybrid market model. The authors divided the identified barriers into two clusters, indicating which of them have been tackled by the proposed market model (Fig. 11).

### 7.1 Strengths of the proposed market model

The proposed market model achieved to tackle a range of barriers within standardisation, lifestyle and administrative categories. The issue of standardisation in terms of market design and market participation starts with the barrier of unclear “Roles and Objectives”. Whereas DSOs see LFMs as interoperable markets for flexibility services requested by DSOs, suppliers and aggregators see them as tools to increase the value of flexibility by making offers on competing markets. The proposed market model clearly states which energy and flexibility services can be achieved by different market-based instruments, focusing on an LFM and its integration with an LEM. Whereas the LFM reflects the flexibility needs of the DSO, the LEM creates value for the end-users. This leads to the next standardisation issue, the “Diversity” of flexibility markets and their different products and access criteria as well as the lack of clear “Business models” for participating in several conflicting markets. The proposed market model follows a price signal-based approach by combining requests from several markets in one single price signal. The numerical examples have shown that relevant revenues can only be achieved by participating on multiple flexibility markets in parallel and the proposed market model provides a suitable framework to optimally offer flexibility for these different needs. However, the major part of cost savings can be achieved through local energy trading rather than the provision of flexibility. Within the lifestyle category, there is a certain “Ignorance” or “Lack of expertise” among end-users, because understanding electricity markets is very complex, making people hesitate to actively participate in such a market. The proposed market model tackles this overarching barrier in two respects. Firstly the end-user can indirectly participate in several markets, but has to commit to a contract only with the LEMO and secondly, the price signal-based approach, translating all energy and flexibility requests into one single price, supports end-users’ understanding and convenience. Consuming energy when prices are low and decreasing the load when prices are high is a straightforward concept that is easy to understand. Moreover, administrative barriers mainly include the “Lack of regulation” for establishing an LFM or P2P trading and the “Charging/cost rules” hampering the implementation of dynamic network tariffs. Therefore, the proposed market model is designed in a way that is flexible and rather agnostic to the specific national legislation. This means that DSOs might either be obliged to meet the flexibility needs by procuring explicit flexibility in a market-based way or the national regulator enables dynamic network tariffs reflecting local flexibility needs.

### 7.2 Weaknesses of the proposed market model

Even though the presented market model manages to overcome a significant amount of barriers, there are still some weaknesses. The authors identified barriers within the lifestyle and administrative categories that cannot be overcome at this stage. Beginning with the lifestyle category, some market participants might still not be interested in taking key decisions about changing their own energy use and therefore not participate in the proposed scheme. This might be because of the still high complexity of the concept, as transparently displaying all the additional revenues and trades leads to increased complexity of the end-users’ invoices, while cost savings might be perceived as too small. Regarding the administrative category, barriers related to regulation aspects cannot be completely overcome at this stage. By unwittingly introducing additional obstacles, national legislation may play a major role in preventing market development features and improvements from being introduced. In order to raise investor interest, the renewable energy sector needs consistent policies and legal procedures (IRENA, 2013). As a consequence, regulatory uncertainty and discontinuity still occur as a result of poorly coordinated policy changes and a complex regulatory system. Moreover, there is also still a lack of transparency with regard to interoperability between different countries with common rules and actors. As a result, there are still some factors that could hinder the adoption and development of such a hybrid market model.

## 8 Conclusion and outlook

In this paper, a user-centric hybrid market model has been proposed to enable the widespread adoption of LFMs and LEMs in the EU.

The results of the Delphi method showed, that for market participants such as DSOs, energy retailers or aggregators, the most critical barriers for such markets arise with regard to standardisation. This includes technical standards, but also the lack of standardised business models and the diversity of flexibility markets.

The proposed hybrid market model has been primarily designed to provide flexibility for the DSO in an LFM, but it also includes an LEM for P2P energy trading among prosumers. The novel approach is that the flexibility is activated through spatio-temporally varying prices in the LEM. This enables flexibility provision on the LFM and also on WS and AS markets through a uniform price signal. Although the main intention is flexibility provision for the DSO, the numerical examples provided in this paper for illustrating the expected financial implications show that the major share of the cost savings comes from P2P energy trading rather than the provision of flexibility services. According to that, end-users’ main profit results from a reduced network tariff for local energy transactions and from the energy prices on the LEM, which is higher than a fixed feed in offering but lower than the supplier price in times of local surplus generation. Adapting the load profiles to these LEM prices creates additional savings, whereas savings achieved through reacting to price signals for flexibility provision towards the DSO or aggregators are only very small. However, it is the strength of the proposed market model to combine several price signals resulting from different energy and flexibility needs. In this way, cost savings can be achieved in a cumulated manner from different markets which do not seem promising as a standalone market, but might be attractive as a combination.

Furthermore, this paper has shown that this novel approach helps to overcome relevant standardisation issues, but also certain barriers regarding end-users’ lifestyles, which is because prices are comprehensible signals that can motivate end-users to participate in such schemes. Barriers that have not been fully tackled and need to be considered for future research include administrative barriers such as an immature national regulation, but also some lifestyle barriers that slow down end-users’ adoption.

The contribution of the proposed market model towards overcoming general regulatory barriers are two-fold. On the one hand, the model is compatible with both explicit flexibility procurement and implicit measures such as adapted network tariffs. On the other hand, questions on the future cost allocation of DSOs remain open for further investigation as well as the effects of this market model in net-metering schemes.

However, the approach of pricing in grid-side flexibility needs in LEM transactions through spatio-temporally varying price signals represents a novel and promising approach for future work. Also, the systematic approach of characterising the market model in this paper offers a valuable framework for future researchers to map their ideas among existing approaches of LFMs and LEMs.

Based on the work presented in this paper, further research activities are planned by testing and evaluating the proposed market model in a real life environment. This will be accomplished within the PARITY project in four pilot sites all around Europe. A final impact assessment will analyse the effects of the hybrid market on grid stability and on the increased deployment of renewable energy sources, in order to facilitate a cleaner and more sustainable energy system.

## CRediT authorship contribution statement

**Guntram Pressmair:** Conceptualization, Methodology, Formal analysis, Data curation, Writing - original draft, Visualization, Project administration. **Evgenia Kapassa:** Conceptualization, Methodology, Formal analysis, Data curation, Writing - original draft, Visualization. **Diego Casado-Mansilla:** Conceptualization, Methodology, Formal analysis, Data curation, Writing - original draft, Visualization. **Cruz E. Borges:** Conceptualization, Data curation, Supervision. **Marinos Themistocleous:** Supervision.

## Declaration of Competing Interest

The authors declare that they have no known competing financial interests or personal relationships that could have appeared to influence the work reported in this paper.

## Appendix Description of the barriers identified: Categories and subcategories

### Current lifestyles

The IPCC (Edenhofer et al., 2011) found that socio-cultural barriers or concerns have different origins and are intrinsically linked to societal and personal values and norms. Such values and norms affect the perception and acceptance of emerging technologies related to energy and the potential impacts of their deployment by individuals, groups and societies. Balta-Ozkan and colleagues (Balta-Ozkan et al., 2013) investigated social barriers to the introduction of smart homes in the UK. In that work, the involved experts identified a lack of fitting to current and changing lifestyles as one of the most important barriers to adopt cutting-edge technologies for the smart homes. This can be clearly brought to the LFM adoption, so this categorisation to understand the social and behavioural aspects that represent a barrier for this emerging market was applied.

Within this category some sub-categories were identified that help to better understand the emerging barriers by giving a context to them.

**Adoption:** Technology adoption is a process. It usually starts with the user becoming aware of the technology and ending with the user embracing the technology and making full use of it. Someone who has embraced a technology is likely to replace the item if it breaks, finds innovative uses for it, and cannot contemplate life without it. Main Barrier Identified (MBI): *“Meeting the evolving needs, demands and preferences of the occupants”*.

**Ideology & involvement:** An ideology is a set of beliefs and values attributed to a person or group of persons. Specifically, a system of ideas and ideals, especially one which forms the basis of economic or political theory and policy. While involvement refers to the willingness of people to participate in the LFM. It is a synonym of engagement. MBI: *“Interdependency among consumers in an individualist society”*.

**Disillusion(ment):** Disappointment resulting from the discovery that something is not as good as one believed it to be. According to the hype cycle, it refers to interest waning as experiments and implementations fail to deliver. Producers of the technology shake out or fail. Investment continues only if the surviving providers improve their products to the satisfaction of early adopters. MBI: *“Smart technology leaves people ‘constantly worrying’ and feeling guilty”*.

**Reluctance and lack of trust:** Unwillingness or disinclination to do something because there is no confidence in the technology. This can be observed for different reasons such as feelings of inequality, resistance to change, fear of digital technologies, an inability to keep up with digital transformation initiatives and disruptions, a feeling of not being taken into account by any given ‘power’, prejudice and the failure of organisations and governments to inform, educate and regulate in a proper and transparent way. MBI: *“Loss of control and apathy, behaviour inertia and fatigue”*.

**Perceived usefulness:** From the Technology Acceptance Model (TAM), this was defined by Fred Davis as “the degree to which a person believes that using a particular system would enhance his or her job performance” (Davis, 1985). It means whether or not someone perceives that technology to be useful for what they want to do. MBI: *“LFM is either little known or still not developed enough to attract the attention of customers”*

**Ignorance/lack of expertise:** The lack of knowledge or information sub-category refers not only to how things work, but even to the very circumstance that things are far more complex than expected. MBI: *“DER or Flexibility markets are too complex for people’s everyday living. They hesitate to understand if their electric power will be ensured”*.

### Administration

This category comprises all barriers that have a relation with legislation, regulation or policy at all levels. This is one of the most diverse categories as barriers of all types can be found among its subcategories.

**Lack of regulation:** this category contains all barriers related to the lack of regulation regarding flexibility markets. Innovation in the energy sector (which is one of the most regulated) is seriously hindered by the slow pace that regulations impose. This is clearly seen in this review as this is one category with the biggest amount of barriers detected. MBI: *“Lack of legislation regarding flexibility markets, Blockchain and energy communities”*.

**Market restrictions:** in opposition to the previous category, this one contains regulations that hinder the possibility of operating a flexibility market. The biggest amount of barriers in this category relates to the restrictions imposed on DSOs and TSOs. MBI: *“DSOs are not allowed to operate freely on the market”*.

**Policy/regulatory incentives:** this category contains the barriers related to the lack of incentives or the unintended effects they have over the deployment of the flexibility market. MBI: *“the lack of funding schemes to deploy the equipment needed”*.

**Charging/cost rules:** this category is related to the previous one, but instead of focusing on the incentives (or lack of them), focus on how actual tariff schemes are a barrier to develop flexibility markets. MBI: *“the different regulations that do not allow to increase peak charges in different countries”*.

**Decision making:** This category reflects all barriers that the different stakeholders face when deciding to participate or deploy a demand response strategy or solution on its company or to their clients. MBI: *“the lack of power of technical staff in companies or the lack of a clear leadership”*.

### Trust

Several studies on technology adoption showed that rather than ‘educating customers’ on the benefits for smart grids, energy demand, etc., industry should focus more on reassuring them that they can and should trust utility companies or vendors. This category refers to the trust among end-users, companies, industry and across all relevant actors and stakeholders. Within this category, the following subcategories emerged from the conducted study:

**Security:** It refers to the defence of digital information and Information Technology (IT) assets against internal and external, malicious and accidental threats. This defence includes detection, prevention and response to threats through the use of security policies, software tools and IT services. MBI: *“cyber-attacks that threaten the smart grid and internet of things (IoT) devices”*.

**Privacy:** Human beings value their privacy and the protection of their personal sphere of life. They value some control over who knows what about them. They certainly do not want their personal information to be accessible to just anyone at any time. But recent advances in information technology threaten privacy and have reduced the amount of control over personal data. MBI: “Combining two sets of innocent data leading to ‘non-innocent’ data”.

**Stakeholders cooperation:** this subcategory refers to the interaction that stakeholders have to hold to provide overall confidence across all involved parties. MBI: *“Imperfect information, asymmetric information, hidden costs, risks”*.

**Communication strategy:**is designed to help end-users to communicate effectively and meet core organisational objectives of the service or good provided. MBI: *“How the recipient of information regards the sender will dictate how such information will be perceived”*.

**Emerging technologies:**are technologies whose development, practical applications, or both are still largely unrealised, such that they are figuratively emerging into prominence from a background of nonexistence or obscurity. MBI: *“Dependency on closed, untrustworthy or unreliable networks, technology and vendors”*.

### Technical

This category comprises all barriers that have a relation with the technical issues that could appear at all levels, from component to overall system. The technical category has been divided in following subcategories:

**System:** this comprises reliability, sustainability, replicability and accuracy of the different digital systems putting in place. MBI: *“The adoption of the most appropriate system based on the end-users’ needs”*.

**Communication:** There are many components (internal and external) that will be part of the final solution and they will have to communicate between them. MBI: *“the security, the balance and the high availability of the network”*.

**Algorithms:** The implementation of new algorithms should overcome some barriers for optimum performance. MBI: *“The lack of historical data to predict the demand”*.

**Deployment:**The deployment of the final system in a test or real scenario could have new barriers such as low speed of the transactions in Blockchain or an increase of resources needed. MBI: *“The integration of these new elements in the existing grid”*.

**Maturity:** Finally, as in the majority of new markets guided by research and innovation, there are some components that are new and the probability of having problems are higher. MBI: *“The need of having more real cases and scenarios to prove the feasibility of the idea”*.

### Standardisation

This category comprises all barriers that have a relation with standardisation at all levels. This is also a diverse category as it comprise technical, legal and administrative barriers among its sub-categories:

**Diversity:** As in all new markets, there is a myriad of technologies and markets schemes proposed or implemented. This category includes the examples found for the flexibility market. MBI: *“Multiple technology requirements in multiple regions”*.

**Interoperability:** As before, given the novelty of the market, no official or de facto standard has emerged, so every solution implements it on technology stack that is not interoperable. MBI: *“The intercommunication problems between the different components in a house or building”*.

**Business models:** The novelty also means that there is not a clear business model for applying these types of markets in several use cases. MBI: *“The conflicts between different markets or the lack of previous experience to assess the participation”*.

**Roles and objectives:** Finally, there is a lack of definition on what a flexibility market should provide to the different stakeholders. MBI: *“There is a mismatch between the idea that policy-makers, energy market participants and end-users have over the expectations of flexibility markets and DERs”*.

### Cost

This category comprises all barriers that are related to the economical cost that customers and companies have to overcome. Cost category have been divided in the following sub-categories:

**Investment:** The initial and maintenance investment that must be done should be taken into account by customers and companies. MBI: *“Hidden costs associated with participation in markets (negotiation and enforcement transaction costs)”*.

**Pricing:**These new markets will carry new contracts and costs related to them. MBI: *“Grid tariffs charged may be higher for LFM members if local grid constraints cannot be avoided persistently”*.

**Margin:**Finally, the expected low margin will make system adoption difficult. MBI: *“A minimum unit size (families) is needed to adopt DR, DER or flexibility benefits”*.

## Footnotes

1 Article 5 of the EU internal electricity market directive (EU, 2019).

2 Which is a requirement in article 4 of the EU internal electricity market directive (EU, 2019).

## References

[b1] Al-Shetwi A.Q., Hannan M., Jern K.P., Mansur M., Mahlia T. (2020). Grid-connected renewable energy sources: Review of the recent integration requirements and control methods. J. Cleaner Prod..

[b2] Asante D., He Z., Adjei N.O., Asante B. (2020). Exploring the barriers to renewable energy adoption utilising multimoora- EDAS method. Energy Policy.

[b3] Bahrami S., Amini M.H. (2018). A decentralized trading algorithm for an electricity market with generation uncertainty. Appl. Energy.

[b4] Balta-Ozkan N., Davidson R., Bicket M., Whitmarsh L. (2013). Social barriers to the adoption of smart homes. Energy Policy.

[b5] Bontius H., Hodemaekers J. (2018). https://www.usef.energy/app/uploads/2018/04/USEF-DSO-WG-report-Overview-Market-based-congestion-management-v1.00-FINAL.pdf.

[b6] Bray, R., 2018. Unlocking Local Energy Markets, Oxford.

[b7] Burger S., Chaves-Ávila J.P., Batlle C., Pérez-Arriaga I.J. (2017). A review of the value of aggregators in electricity systems. Renew. Sustain. Energy Rev..

[b8] Burger S.P., Jenkins J.D., Batlle C., Perez-Arriaga I.J. (2019). Restructuring revisited part 2: Coordination in electricity distribution systems. Energy J..

[b9] CEER (2018). https://www.ceer.eu/documents/104400/-/-/e5186abe-67eb-4bb5-1eb2-2237e1997bbc.

[b10] CEER (2020). https://www.ceer.eu/documents/104400/-/-/fd5890e1-894e-0a7a-21d9-fa22b6ec9da0.

[b11] CEER (2020). https://www.ceer.eu/documents/104400/-/-/e436ca7f-a0df-addb-c1de-5a3a5e4fc22b.

[b12] Davis F.D. (1985).

[b13] Edenhofer O., Pichs-Madruga R., Sokona Y., Seyboth K., Kadner S., Zwickel T., Eickemeier P., Hansen G., Schlömer S., von Stechow C. (2011).

[b14] 2019. Day-Ahead Prices, https://transparency.entsoe.eu/.

[b15] Prices of activated balancing energy, https://transparency.entsoe.eu/.

[b16] ENTSO-E O., ebIX R., EFET Y. (2020). https://www.entsoe.eu/Documents/EDI/Library/HRM/Harmonised_Role_Model_2020-01.pdf.

[b17] Esmat A., Usaola J., Moreno M.a. (2018). Distribution-level flexibility market for congestion management. Energies.

[b18] EU A. (2018).

[b19] EU A. (2019).

[b20] European Commission (2020). https://eur-lex.europa.eu/legal-content/EN/TXT/?uri=CELEX:52020DC0562.

[b21] Frieden D., Tuerk A., Roberts J., d’Herbemont S., Gubina A. (2019). https://www.compile-project.eu/wp-content/uploads/COMPILE_Collective_self-consumption_EU_review_june_2019_FINAL-1.pdf.

[b22] Good N., Ellis K.A., Mancarella P. (2017). Review and classification of barriers and enablers of demand response in the smart grid. Renew. Sustain. Energy Rev..

[b23] Hamwi M., Lizarralde I., Legardeur J. (2020). Demand response business model canvas: A tool for flexibility creation in the electricity markets. J. Cleaner Prod..

[b24] Heinrich C., Ziras C., Syrri A.L., Bindner H.W. (2020). Ecogrid 2.0: A large-scale field trial of a local flexibility market. Appl. Energy.

[b25] Ibn Saif A.U.N., Khadem S.K. (2020). 2020 17th International Conference on the European Energy Market (EEM).

[b26] IRENA, ., 2013. Overcoming Barriers to Authorizing Renewable Power Plants and Infrastructure. In: IRENA Executive Strategy Workshop on Renewable Energy in South East Europe, https://www.irena.org/-/media/Files/IRENA/Agency/Events/2013/Jan/12_1/Background_Paper-D.pdf?la=en&hash=CAE94D402BD2800E38B02F1F45D8833AE64C2D1D.

[b27] Jin X., Wu Q., Jia H. (2020). Local flexibility markets: Literature review on concepts, models and clearing methods. Appl. Energy.

[b28] Klaassen E., Van der Laan M. (2019). https://www.usef.energy/app/uploads/2019/02/USEF-White-Paper-Energy-and-Flexibility-Services-for-Citizens-Energy-Communities-final-CM.pdf.

[b29] Kouzelis K., Bak-Jensen B., Pillai J.R. (2015). 2015 IEEE Power & Energy Society Innovative Smart Grid Technologies Conference (ISGT).

[b30] Linstone H.A., Turoff M. (1975).

[b31] Mendes G., Ferreira J.R., Albuquerque S., Trocato C., Kilkki O., Repo S. (2019). https://www.cired-repository.org/handle/20.500.12455/93.

[b32] Mengelkamp E., Gärttner J., Rock K., Kessler S., Orsini L., Weinhardt C. (2018). Designing microgrid energy markets. Appl. Energy.

[b33] Mengelkamp E., Schlund D., Weinhardt C. (2019). Development and real-world application of a taxonomy for business models in local energy markets. Appl. Energy.

[b34] Mihaylov M., Razo-Zapata I., Nowé A. (2018). Transforming Climate Finance and Green Investment with Blockchains.

[b35] Minniti S., Haque N., Nguyen P., Pemen G. (2018). Local markets for flexibility trading: Key stages and enablers. Energies.

[b36] NODES (2018). https://nodesmarket.com/download/whitepaper/.

[b37] Olivella-Rosell P., Bullich-Massagué E., Aragüés-Peñalba M., Sumper A., Ottesen S.Ø., Vidal-Clos J.-A., Villafáfila-Robles R. (2018). Optimization problem for meeting distribution system operator requests in local flexibility markets with distributed energy resources. Appl. Energy.

[b38] Olivella-Rosell P., Lloret-Gallego P., Munné-Collado Í., Villafafila-Robles R., Sumper A., Ottessen S., Rajasekharan J., Bremdal B. (2018). Local flexibility market design for aggregators providing multiple flexibility services at distribution network level. Energies.

[b39] PARITY (2020). https://parity-h2020.eu/about.

[b40] Radl J., Fleischhacker A., Revheim F.H., Lettner G., Auer H. (2020). Comparison of profitability of PV electricity sharing in renewable energy communities in selected European countries. Energies.

[b41] Ramos A., De Jonghe C., Gómez V., Belmans R. (2016). Realizing the smart grid’s potential: Defining local markets for flexibility. Util. Policy.

[b42] Repo, S., Kilkki, O., Annala, S., Terras, J.M., Almeida, B., 2020. Local flexibility market balance settlement, Berlin.

[b43] Sajjadi S.M., Mandal P., Tseng T.-L.B., Velez-Reyes M. (2016). 2016 North American Power Symposium (NAPS).

[b44] Schittekatte T., Meeus L. (2020). Flexibility markets: Q&A with project pioneers. Util. Policy.

[b45] Seetharaman T., Moorthy K., Patwa N., Saravanan M., Gupta Y. (2019). Breaking barriers in deployment of renewable energy. Heliyon.

[b46] Siano P., De Marco G., Rolan A., Loia V. (2019). A survey and evaluation of the potentials of distributed ledger technology for peer-to-peer transactive energy exchanges in local energy markets. IEEE Syst. J..

[b47] van Soest H. (2018). Peer-to-peer electricity trading: A review of the legal context. Compet. Regul. Netw. Ind..

[b48] Spiess T., De Sousa C. (2016). Barriers to renewable energy development on brownfields. J. Environ. Policy Plan..

[b49] Stanley R., Johnston J., Sioshansi F. (2019). Consumer, Prosumer, Prosumager.

[b50] Torbaghan S.S., Blaauwbroek N., Kuiken D., Gibescu M., Hajighasemi M., Nguyen P., Smit G.J., Roggenkamp M., Hurink J. (2018). A market-based framework for demand side flexibility scheduling and dispatching. Sustain. Energy Grids Netw..

[b51] USEF (2015). https://www.usef.energy/download-the-framework/.

[b52] Villar J., Bessa R., Matos M. (2018). Flexibility products and markets: Literature review. Electr. Power Syst. Res..

[b53] Weinhardt C., Mengelkamp E., Cramer W., Hambridge S., Hobert A., Kremers E., Otter W., Pinson P., Tiefenbeck V., Zade M. (2019). Proceedings of the Tenth ACM International Conference on Future Energy Systems.

[b54] Yu-zhuo Z., Xin-gang Z., Ling-zhi R., Yi Z. (2017). The development of the renewable energy power industry under feed-in tariff and renewable portfolio standard: A case study of China’s wind power industry. J. Cleaner Prod..

[b55] Zabaleta K., Casado-Mansilla D., Kapassa E., Borges C.E., Preßmair G., Themistocleous M., López-de Ipiña D. (2020). 2020 5th International Conference on Smart and Sustainable Technologies (SpliTech).

[b56] Zhang C., Ding Y., Nordentoft N.C., Pinson P., stergaard J. (2014). FLECH: A danish market solution for DSO congestion management through DER flexibility services. J. Mod. Power Syst. Clean Energy.

[b57] Zhang C., Wu J., Long C., Cheng M. (2017). Review of existing peer-to-peer energy trading projects. Energy Procedia.

[b58] Zou Y., Zhao J., Gao X., Chen Y., Tohidi A. (2020). Experimental results of electric vehicles effects on low voltage grids. J. Cleaner Prod..

